# Inter-assessor reliability of practice based biomechanical assessment of the foot and ankle

**DOI:** 10.1186/1757-1146-5-14

**Published:** 2012-06-20

**Authors:** Hannah L Jarvis, Christopher J Nester, Richard K Jones, Anita Williams, Peter D Bowden

**Affiliations:** 1 School of Health Sciences, Centre for Health Sciences Research, University of Salford, Salford, M6 6PU, UK

## Abstract

**Background:**

There is no consensus on which protocols should be used to assess foot and lower limb biomechanics in clinical practice. The reliability of many assessments has been questioned by previous research. The aim of this investigation was to (i) identify (through consensus) what biomechanical examinations are used in clinical practice and (ii) evaluate the inter-assessor reliability of some of these examinations.

**Methods:**

Part1: Using a modified Delphi technique 12 podiatrists derived consensus on the biomechanical examinations used in clinical practice. Part 2: Eleven podiatrists assessed 6 participants using a subset of the assessment protocol derived in Part 1. Examinations were compared between assessors.

**Results:**

Clinicians choose to estimate rather than quantitatively measure foot position and motion. Poor inter-assessor reliability was recorded for all examinations. Intra-class correlation coefficient values (ICC) for relaxed calcaneal stance position were less than 0.23 and were less than 0.14 for neutral calcaneal stance position. For the examination of ankle joint dorsiflexion, ICC values suggest moderate reliability (less than 0.61). The results of a random effects ANOVA highlight that participant (up to 5.7°), assessor (up to 5.8°) and random (up to 5.7°) error all contribute to the total error (up to 9.5° for relaxed calcaneal stance position, up to 10.7° for the examination of ankle joint dorsiflexion). Kappa Fleiss values for categorisation of first ray position and mobility were less than 0.05 and for limb length assessment less than 0.02, indicating slight agreement.

**Conclusion:**

Static biomechanical assessment of the foot, leg and lower limb is an important protocol in clinical practice, but the key examinations used to make inferences about dynamic foot function and to determine orthotic prescription are unreliable.

## Background

Abnormal foot and ankle biomechanics are implicated in a wide range of foot and lower limb disorders managed by podiatry and other allied health professions [1]. Exercise and foot orthotic treatment approaches are commonly based on an assessment of a patient’s foot biomechanics [2]. Protocols for clinical assessment of foot biomechanics are broadly based on those advocated by Root et al. [3,4] and more recent literature [5,6]. Root et al. [3,4] proposed a conceptual framework describing normal and abnormal foot function during walking and an assessment protocol that enables a clinician to predict the function of the foot during walking via a static (i.e. standing or non weight bearing) assessment of the foot (the Root static foot assessment protocol). Understanding the reliability of an assessment protocol aims to identify whether examinations are consistent between assessors and across time [7] (when there is no change in the status of the foot). Good reliability is the basis for sound professional practice and is essential for quantifying the value of an examination [7]. There is already evidence that some or all static foot assessment protocols are unreliable [8-12]. However, most studies have tested only part of the assessment protocol described by Root et al. [3] and have largely adopted the examinations as they were first described [13]. In reality the current implementation of the protocol for static foot assessment is influenced by many factors, including national or local professional knowledge (via discussion at workshops/conferences), clinical experience (clinicians would adapt their practice to their learning), and practical constraints (time available for an assessment, the range of orthotic prescriptions available to a clinician, and the particular profile of patients the clinician sees in their practice). Thus, the reliability of static foot assessment protocols as they are currently used in practice has not been evaluated.

Relaxed and ‘neutral’ calcaneal stance position (RCSP and NCSP) are arguably the core elements of the Root et al. [3] static foot assessment and directly influence orthotic prescription. Their importance to practice is reflected in the fact that they have been subject to considerable scrutiny by the physical therapy and related communities [8-14]. Menz [8] highlighted how the assessment is prone to erroneous subjectivity due to skin movement artefact, pen marker thickness and practitioner dexterity. Menz and Keenan [9] examined the inter-assessor reliability of a gravity angle finder to measure NCSP and RCSP. Pearson’s correlation coefficient values (and standard error of measurement (SEM)) were 0.367 (±3.77°) and 0.742 (±6.27°) respectively. Use of a digital goniometer did not significantly improve measurements, with correlation (r) values of 0.558 (±8.47°) and 0.742 (±6.47°) respectively. Keenan and Bach [12] reported 95 % confidence intervals of 5.1° (range −9.0° to 7.0°) for RCSP and 4.1° (range −2.0° to 13.0°) for NCSP over two measurement sessions. Both studies conclude that the large variation between assessors would affect diagnosis and treatment rationale.

Rome [15] highlighted the difficulty of assessing the sagittal plane motion of the ankle joint. The poor alignment of the goniometer, non-identification of bony landmarks and the variation in force applied would all contribute to error [15]. Elveru et al. [16] recorded an ICC value of 0.50 and Jonson and Gross [1] an ICC of 0.65 when examining the inter-assessor reliability of assessing ankle joint dorsiflexion with a goniometer. The greater reliability in the latter study may be due to Jonson and Gross [1] allowing participants to maximally dorsiflex their foot rather than a clinician manipulate the foot.

The measurement of first ray mobility and position has been measured directly (e.g. mm) and categorically (e.g. classification of the range of motion or the position of the first ray). Glascoe et al. [17] reported very poor inter-assessor reliability for the direct measurement of first ray mobility using a ruler, with an ICC value of 0.05. Similarly Cornwall et al. [18] observed poor agreement and inter-assessor reliability for the classification of first ray mobility, with only 12.5 % agreement for classification of first ray mobility as hypomobile and 25.0 % agreement for hypermobile.

There are two approaches to limb length examination: direct measures (e.g. tape measure), [19,20] and in-direct methods such as palpation of bony pelvic landmarks and placing blocks under the heel of the participant [21]. The latter appear to have greater reliability [21]. Woerman and Binder-MacLeod [21] recorded small mean differences (less than 4.3 mm) across five assessors when palpating the iliac crest and placing small blocks under the heel of the participant to measure the differences in limb length. Jonson and Gross [1] recorded good inter-assessor reliability (ICC = 0.70) when placing blocks under the heel and using a levelling device to ascertain pelvis obliquity.

Understanding how foot biomechanics are assessed in current practice, and the reliability of the assessments, enables us to understand: (1) whether current practices have changed since Root et al. [3] first introduced their work; and (2) the credibility of the assessment protocols used in current practice. This project aimed to: (i) identify (through consensus) what biomechanical examinations are used in clinical practice and (ii) evaluate the inter-assessor reliability of a subset of these assessments.

## Methods

### Part 1 Identification of biomechanics assessment protocols used in practice

Twelve podiatrists (working in state funded and private health care settings, six male, mean age 42) specialising in foot and ankle biomechanics were invited to participate. All worked within a specialist biomechanics/musculoskeletal clinic and had at least 3 years clinical experience at this specialist level. Ethical approval was granted (University of Salford Institutional Committee) and all participants gave written consent. A Delphi method [22] was chosen to derive consensus on a foot biomechanics assessment protocol. The Delphi method [22] is a systematic and structured examination technique involving a panel of experts. The method combines use of questionnaires and group discussion to derive consensus [22].

There were three keys phases to the development of a consensus.

Phase 1: Questionnaire. All podiatrists answered a questionnaire (Additional file 1) anonymously and without discussion. The questionnaire (written by HJ and PB) investigated the use of static foot, leg and lower limb biomechanical examinations and gait analysis protocols by each podiatrist. Questions were derived from Root et al. [3], current undergraduate syllabus, information from Valmassey [23] and Michaud [24]. There was also space provided for podiatrists to report any additional examinations used.

Most questions required Yes/No answers and required information on how often each examination was used, the method and whether the information was used to classify foot type and/or to develop a treatment rationale.

Phase 2: Development of draft consensus from results of the questionnaire. From the completed questionnaires, PB and HJ identified where there was both agreement and disagreement amongst the expert panel. Agreement existed when there was an identifiable trend amongst podiatrists, for example the majority of podiatrists used the same measurement technique. Disagreement was where there was poor consensus between podiatrists, for example less than half used a particular examination. A separate adjudicator (CN) was present throughout. A draft assessment protocol was developed based on the questionnaire responses.

Phase 3: Group discussion. A group discussion (led by PB, HJ took notes) explored the validity of the questionnaire results and draft foot assessment protocol from Phase 2. Discussion orientated around whether it was true reflection of the current practice of the panel members but also related professional disciplines. The areas of agreement and disagreement from the questionnaire results were elaborated upon though open discussion. Podiatrists explained in more detail their assessment methodology, their conceptual understanding of the normative basis to which pathological cases are compared and the rationale for their assessment plan.

### Part 2 Evaluation of the inter-assessor reliability of the biomechanical assessment protocol

Eleven podiatrists (working in state funded and private health care settings, six male, mean age 46) specialising in foot and ankle biomechanics practice volunteered to participate. All worked within a specialist biomechanics/musculoskeletal clinic and had at least 5 years clinical experience at this specialist level.

Each podiatrist assessed six asymptomatic participants (three male, mean age 25, mean body mass index [BMI] 23), using a subset of the assessment protocol defined in Part 1 of the study. Ethical approval was granted from the University of Salford Institutional Committee and all participants gave written consent. This investigation was conducted nine months after Part 1.

Four of the eight biomechanical examination procedures identified in Part 1 were selected for the inter-assessor reliability study. These were selected primarily because the podiatrists identified them as essential rather than optional components of their clinical assessment. However, they also provided some assessment of the lower limb as well as the foot and could be completed within a reasonable time frame. The four assessments selected by podiatrists were used for all or the majority of patients and provided information critical to the development of treatment rationale and orthotic prescription. Thus the four selected contributed more to clinical practice than the four assessments omitted.

The assessments used in the inter-assessor study were 1) NCSP and RCSP, 2) ankle joint range of dorsiflexion, 3) first ray mobility and position, and 4) limb length examination. They were assessed quantitatively or qualitatively according to the preferences identified in Part 1.To help maintain consistency in how the 11 podiatrists implemented the assessment protocol, an information sheet and demonstration was provided. The participants whose feet were to be assessed were placed in six separate cubicles at the University clinic. Assessments were conducted as per the protocols described in Table 1 and podiatrists were allocated 30 minutes to assess each participant and at least 30 minutes rest between each assessment. In accordance with clinical practice, each assessment was completed once for each foot. Each podiatrist recorded their assessments in a booklet. No discussion was allowed between podiatrists or participants during the assessments. All pen marker lines on the participants were removed between podiatrists.

**Table 1:** The examination methods used by podiatrists in current practice (identified from Phase 1,2 and 3)

**Biomechanical examination**	**Method**
Neutral calcaneal stance position (NCSP) and relaxed calcaneal stance position (RCSP)	(i) Participant standing (ii) Position both feet into NCSP (iii) Pen marker bisection line drawn onto the posterior aspect of the calcaneus on both feet (iv) Measurement recorded using digital biometer for right foot (v) Identify if calcaneus is positioned varus or valgus (vi) Repeat procedure with left foot (vii)Both feet resume RCSP, measurement of the bisection line using a digital biometer for both feet
Range of ankle joint dorsiflexion	(i) Participant supine and sitting with back straight against plinth (ii) A straight reference line is drawn onto the lateral aspect of leg indicating where one of the tractograph arms should be positioned (iii) Tractograph is positioned with one lever arm running parallel to the lateral aspect of leg and the other positioned parallel to the plantar aspect of the foot running distally (iii) With the knee joint extended, the foot is maximally dorsiflexed and the measurement on the tractograph recorded (iv) The knee joint is held in a flexed position, the ankle joint is maximally dorsiflexed and the measurement on the tractograph recorded (v) Repeat procedure with other foot
Position and mobility at the first ray	(i) Participant supine and sitting with back straight against plinth (ii) First ray position classification (dorsiflexed/plantarflexed or neutral) (iii) First ray mobility classification (flexible/rigid/normal) (iv) Repeat procedure with cother foot
Forefoot to rearfoot relationship (frontal plane)	(i) Participant prone, lying down (ii) Raise one side of pelvis from couch with a cushion/pillow, so that the long axis of the contra lateral foot is vertical (iii) Position the foot in subtalar joint neutral (iv) Visually observe position of forefoot relative to rearfoot. Categorise if neutral/everted/inverted.(v) Repeat procedure with other foot
Range of motion at the first MTPJ	(i) Participant supine and sitting with back straight against plinth with legs extended in front (ii) Place arms of the goniometer parallel to the long axis of the first metatarsal and the proximal phalanx of the hallux (iii) Manually dorsiflex first MTPJ with first ray free to move and measure range of motion with goniometer (iv) Repeat stage 3, with first ray held in a neutral position (v) Repeat procedure with left foot
Foot Posture Index	The 6 Point FPI is to be used and follows the protocol described by Redmond et al. [31]
Limb length examination	(i) Participant standing in RCSP (ii) Both ASIS are palpated, identification of whether a limb length discrepancy is present (iii) Classification of which leg is longer and whether this is less than 5 mm, more than 5 mm or more than 10 mm
Visual gait analysis	On conducting a clinical gait analysis, key determinants to be observed (i) Position of foot at heel strike (ii) Forefoot and midfoot position during loading phase. (iii) Foot position and motion during propulsion and re-supination (iv) Movement of the foot and leg during swing phase (v) Motion of the hip and knee (vi) Timing and magnitude of motion

### Statistical analysis

The researchers were blind to the data in each booklet. All data was collated into Microsoft Excel and then processed through Statistical Package Social Science Software (Version 17.0) (SPSS, Chicago, Illinois, USA). The mean, range, standard deviation (SD) and 95 % confidence intervals (95 % CI) were calculated for NCSP, RCSP and the range of ankle joint dorsiflexion.

Inter-assessor reliability for RCSP, NCSP and the range of ankle joint dorsiflexion were calculated using ICC (2,1) in accordance with Rankin and Stokes [25]. ICC values were chosen as they assess the consistency of quantitative measurements made by multiple testers (clinicians) measuring the same objects (participants) [7]. Bruton et al. [7] suggest that ICC values should not be interpreted clinically in isolation. Therefore a random effects ANOVA (analysis of variance, crossed random effects model) [26] was used to enable further evaluation of reliability. A random effects ANOVA models y as a constant, plus a random effect due to the assessor (clinician), a random effect due to the participant (e.g. moved their feet) and an overall random error of the examination itself. (E ¹ assessor error, E ² participant error, E random error).

(1) $$\text{y}=\mu +\sqrt{{\text{E}}^{1}}+{\text{E}}^{2}+\text{E}$$

This calculates the extent of between participant variability, between assessor variability and the amount of random error in the examination. This provides an indication of where the majority of error occurs. Therefore for each part of the assessment (e.g. NCSP, RCSP), the error variables have to be accounted for in addition to the true value of the feature being assessed:

Value provided by the assessor = actual value + (assessor error (E ¹) + participant error (E ² ) + random error (E)).

A particular advantage of the random effects ANOVA is that the outcomes are expressed in the same units as the measurement and thus are easily interpreted in terms of clinical practice. In addition, the three sources of error can be combined to provide an indication of the total error due to participant, assessor and random error:

Total error = √(assessor error (E ¹) + participant error (E ² ) + random error (E).

The assessment of first ray mobility and position and limb length involved categorical data, therefore the percentage agreement (%) [27] and a Fleiss Kappa [28,29] were chosen.

Percentage agreement can lack sensitivity as it does not adjust for that agreement occurring by chance [27]. A Fleiss Kappa calculates the reliability of agreement between a fixed number of assessors [28-30] and is a better representation of true inter-assessor reliability [27]. Fleiss Kappa values range from <0 for poor agreement to 1.00 for perfect agreement [28,30,31]. Both of these statistical measures are consistent with the available literature [17,18].

## Results

### Part 1

Tables 1 and 2 represent the results of the questionnaire and the group discussion. Three key trends were derived from the questionnaire (Phases 1 and 2) and formed the basis to the subsequent discussion (Phase 3). These were:

(i) The main basis to biomechanical assessment of the foot and ankle is the description provided by Root et al. [3,4].

(ii) Podiatrists “estimate” rather than measure foot or limb position and motion.

(iii) In addition to their static assessment, podiatrists conduct a dynamic gait assessment focusing on observation at key events of the gait cycle.

**Table 2:** Results of Phase 1, 2 (questionnaires) and 3 (group discussion) on assessment of the foot and ankle

**Biomechanical examination**	**No. of podiatrists that use the assessment (total =12)**	**Key features of examination (derived from questionnaire)**	**Consensus from group discussion**
NCSP and RCSP	9	Position is estimated not measured 9/9 Use this as an assessment of foot type 8/9 Use this to develop a treatment rationale (for example orthotic prescription)	Frontal plane position of the calcaneus relative to the leg was always observed Foot type is classified as pronated/supinated/neutral This is a key biomechanical examination of the foot Heel bisection lines do not add value to the examination Podiatrists feel that they could accurately measure the frontal plane position of the calcaneus quantitatively if required
Range of motion at the ankle joint	12	Range of motion is estimated, not measured 12/12 podiatrists assessed with the knee extended 9/12 podiatrists assessed with the knee flexed The total range of motion and range of dorsiflexion are assessed	Podiatrists state that the normal range of ankle dorsiflexion is 10° Assessment of the range of motion is based on the podiatrist's own experience as to what they perceive as normal and not through the use of a goniometer/other measuring device Podiatrists feel that they could accurately measure the range of ankle joint dorsiflexion quantitatively if required
Range of motion at the subtalar joint	11	Motion is estimated not measured Subtalar joint neutral (non weight-bearing) is used as a reference position to determine the amount of pronation and supination	Podiatrists believe that this examination is a good indicator of dynamic foot function, but it is difficult to conduct
Position and mobility of the first ray	11	Position and mobility are estimated not measured 9/12 use categorical rather than numerical data	Consensus from podiatrists was that for examination of first ray mobility and position categorical data (e.g. “rigid/flexible/normal”) is more useful than numerical data
Forefoot to rearfoot relationship	11	Position is estimated not measured. 11/11 use this assessment in the frontal plane only	No consensus on what should be used to define the forefoot (e.g. use middle three metatarsals or use all five metatarsals)
Range of motion at the first MTPJ	11	Motion is estimated not measured 9/12 assess the total range of motion of the first MTPJ 6/12 assess the range of first MTPJ dorsiflexion	Consensus from podiatrists was that assessment of the forefoot was dependent on the presenting musculoskeletal complaint/injury and their focus was always on the function of the rearfoot
Foot Posture Index (FPI) [31]	6	6/12 use the FPI as an assessment of foot type/posture	Some podiatrists were unaware of the FPI Some podiatrists did use individual elements of FPI
Assessment of the lower limb	12	All podiatrists assess the lower limb, leg and foot	Podiatrists state that it is important to assess the pelvis, lower limb, leg and foot in a biomechanical assessment
Leg length discrepancy examination	7 to 9	Limb length is estimated not measured 9/12 assess anatomical limb length 7/12 assess functional limb length	Consensus from podiatrists was that the examination of limb length is important and a limb length discrepancy is a common cause of abnormal biomechanical function of the foot, leg and lower limb Podiatrists feel that the process of obtaining a precise measurement (through tape measure) is not reliable and instead categorise the leg length discrepancy, for example <5 mm, <10 mm, >10 mm Measurement of limb length should also involve shoulder tilt, ASIS symmetry (supine and standing)
Additional biomechanical examinations	NA	Examination of internal and external hip rotation Examination of hamstring flexibility (Straight leg raise test) "Heel raise" test to assess function of tibialis posterior	Podiatrists state that these are not mandatory examinations and therefore are only used for specific clinical presentations
Gait Analysis	11	11/11 assess the dynamic function of the foot, ankle and knee 10/11 assess the dynamic function of the hip and upper body	Dynamic assessment is as important as static examination for diagnosis and development of a treatment plan
Key determinants of the gait cycle to be observed during a routine gait analysis	NA	Position of foot at heel strike Forefoot and midfoot position during loading phase.Foot position and motion during propulsion and re-supination Movement of the foot and leg during swing phase Motion of the hip and knee Timing and magnitude of motion 4 to 6/12 podiatrists had access to gait analysis equipment e.g. pressure plate, 2D video analysis	Podiatrists state that they follow a relatively consistent protocol when conducting a clinical gait analysis assessment. The protocol involved identifying foot function at key events during the gait cycle and always aiming to analyse these from a visual perspective Consensus among podiatrists was that they would compare the dynamic function of a patient’s foot and ankle to the description of “normal” they were taught at undergraduate level, the predominant basis for this was Root et al. [3,4] The consensus among podiatrists was that additional gait analysis equipment did not aid their assessment or treatment plan. All podiatrists felt they were confident in their visual analysis of the patient walking and what was feasible within the time constraints

The biomechanical assessment protocol identified through consensus comprised the following:

(i) Examination of the foot in relaxed and neutral calcaneal stance position (RCSP and NCSP)

(ii) Examination of forefoot to rearfoot relationship in the frontal plane

(iii) Examination of the range of ankle joint dorsiflexion

(iv) Examination of the position and mobility of the sagittal plane motion at the first ray

(v) Examination of the range of sagittal plane motion at the first metatarsophalangeal joint (MTPJ)

(vi) Foot Posture Index [31]

(vii) Examination of limb length

(viii) Visual gait analysis. Table 1 describes the protocols for the assessments chosen.

### Part 2

The results indicate poor inter-assessor reliability for the four examinations. Table 3 displays the reliability results for RCSP and NCSP. For RCSP an ICC of 0.23 (right), 0.14 (left) and 0.14 (right) and 0.11 (left) for NCSP suggest poor inter-assessor reliability. All mean 95 % CI were above 3.7° and the mean range of NCSP and RCSP values were greater than 8.8° (Table 4). The results of the random effects ANOVA indicate that the greatest error was random error (up to 4.9°), while the assessor error was up to 3.4°.

**Table 3:** Inter-assessor reliability results for all examinations

**ICC values for RCSP and NCSP**		
	RCSP (ICC 2’1)	NCSP (ICC 2’1)
Right foot	0.23	0.14
Left foot	0.14	0.11
ICC values for the range of ankle joint dorsiflexion		
	Knee extended	Knee flexed
Right foot	0.44	0.61
Left foot	0.42	0.51
Fleiss Kappa values for categorisation of first ray position and mobility		
	First ray position	First ray mobility
Right foot	−0.03	0.05
Left foot	0.01	−0.01
Fleiss Kappa values for the categorisation of limb length examination		
	Identification of longer leg	Identification of longer leg length
Limb length examination	0.02	0.02

**Table 4:** Descriptive analysis of the variation between assessors in the examination of RCSP and NCSP

**Foot**	**Examination**	**Mean (°)**	**SD (°)**	**Range (°)**	**95 % CI (°)**
Right foot	RCSP	0.2	3.2	11.2	−2.0 to 2.6
	NCSP	3.4	3.6	12.2	0.9 to 5.8
Left foot	RCSP	−0.4	3.4	11.2	−2.7 to 1.9
	NCSP	3.2	2.8	8.8	1.2 to 4.9
Results of random effects ANOVA					
		√Estimate of covariance parameter (°)			
Foot	Examination	√E random error (°)	√E ¹ assessor error (°)	√E ² subject error (°)	Total (°)
Right foot	RCSP	3.2	0.6	1.8	3.8
	NCSP	2.2	2.9	0.8	3.8
Left foot	RCSP	4.9	3.4	1.1	9.5
	NCSP	2.2	1.8	1.0	3.1

Table 3 demonstrates ICC values for the examination of the range of ankle joint dorsiflexion. There was moderate agreement with 0.44 (right) and 0.42 (left) for knee extended and 0.61 (right) and 0.51 (left) for knee flexed. All mean 95 % CI were above 9.0°, and the mean range of ankle dorsiflexion values was greater than 20.5° (Table 5). The results of the random effects ANOVA indicate that there were comparable contributions from the three sources of error, with values ranging from 4.3° to 5.8°.

**Table 5:** Descriptive analysis of the variation between assessors in the examination of the range of ankle joint dorsiflexion

Foot	Examination	Mean (°)	SD (°)	Range (°)	95 % CI (°)
Right foot	Knee Extended	3.9	7.0	23.0	−0.8 to 8.6
	Knee Flexed	10.5	7.3	23.0	5.6 to 15.5
Left foot	Knee Extended	3.0	6.6	20.5	0.1 to 9.1
	Knee Flexed	7.5	6.9	22.2	5.2 to 14.2
Results of random effects ANOVA					
		√Estimate of covariance parameter (°)			
Foot	Examination	√E random error (°)	√E ¹ assessor error (°)	√E ² subject error (°)	Total error (°)
Right foot	Knee Extended	5.2	4.9	4.6	10.7
	Knee Flexed	4.5	5.8	5.7	9.3
Left foot	Knee Extended	4.9	4.6	4.3	8.0
	Knee Flexed	5.1	4.9	5.2	8.7

The results for classification of first ray position and mobility are displayed in Table 3 and Table 6. There was greater consistency for the categorisation of mobility compared to first ray position. Fleiss Kappa values of −0.03 (right foot) and 0.01 (left foot) for categorisation of position and for the range of first ray motion (0.05 (right) and −0.01 (left)).

**Table 6:** Descriptive analysis of the variation between assessors in the categorisation of first ray position and mobility

Percentage agreement values				
Foot	Examination	Plantarflexed (%)	Neutral (%)	Dorsiflexed (%)
Right foot	First ray position	55.0	31.5	13.5
Left foot		62.0	30.0	8.0
		Flexible (%)	Neutral (%)	Rigid (%)
Right foot	First ray mobility	94.0	1.5	4.5
Left foot		91.0	7.0	2.0

Table 3 and 7 demonstrates the results for examination of limb length. There was less agreement on the size of the difference in limb length than the identification of the longer limb when evaluating the percentage agreement values, however results were comparable according to Fleiss Kappa values (0.02 for both longer leg and the difference in leg length). Clinicians consistently reported differences in limb length of 5 mm or less (Table 3 and 7).

**Table 7:** Descriptive analysis of the variation between assessors for the categorisation of limb length examination

Percentage agreement values				
Examination	Right (%)	Left (%)	None (%)	
Identification of longer leg	64.0	12.0	24.0	
	up to 5 mm (%)	5-10 mm (%)	greater than 10 mm (%)	None (%)
Identification of longer leg length	23.0	39.0	14.0	24.0

## Discussion

### Biomechanical assessment protocol that podiatrists use in clinical practice

The assessment protocol developed in Part 1 of this investigation is largely a modified version of Root et al. [3]. The description provided by Root et al. [3,4] is still very much at the forefront of clinical assessment of foot biomechanics and the basis for clinical descriptors of foot function during gait. This demonstrates the continued influence of Root et al. [3,4] and the strong effect undergraduate education has on subsequent practice. However, the inclusion of the Foot Posture Index [31] and use of visual gait assessment signify that podiatrists have adopted new assessment approaches that they deem to add value. This did not extend as far as the use of potentially valuable instrumented gait assessment methods (For example video analysis, pressure plate).

Contrary to the specific instructions of Root et al. [3], podiatrists choose to estimate and classify joint position/motion rather than ascertain a directly measured numerical value. For example, when assessing the ankle joint, podiatrists choose to estimate the range of dorsiflexion rather than use a goniometer. Podiatrists felt that their experience was sufficient to accurately classify the range of motion as normal, excessive or restricted. All podiatrists stated that they were confident this approach was valid and cited time constraints as the primary barrier to use of objective measures. However, continuing to use assessments that have been shown to have low reliability is likely to be considered unsound practice. If reliability could be improved by an objective rather than subjective assessment, even if it takes longer to complete, then this could form a strong case to extend the time available for the assessment of patients.

These differentiations from the original description and instructions of Root et al. [3,4] justify the consensus exercise in Part 1 and ensure that our investigation of inter-assessor reliability is relevant to current clinical practice.

### Inter-assessor reliability

There was poor inter-assessor reliability recorded for all of the static biomechanical examinations of the foot, leg and lower limb which questions their value in clinical practice. RCSP and NCSP produced poor inter-assessor reliability results (all ICC values were less than 0.23), and this concurs with the available literature. Picciano et al. [32] recorded ICC values for NCSP of 0.15 and 95 % confidence intervals of 0.87° to 8.65°. The results of the random effects ANOVA suggest that for RCSP and NCSP random error is the key issue. Differences between assessors of this scale would create different treatment plans and orthotic designs. Both Menz [8] and Elveru et al. [16] highlight that an overwhelming priority is placed upon the outcomes of these measurements in clinical assessment and orthotic prescription. However, the poor reliability and large variation in the results recorded here and elsewhere [8,9,12,16] should be clinically unacceptable and we therefore question their continued use in clinical practice [8,15,16].

Although podiatrists reported some difficulty in using the goniometer [15], moderate reliability was observed for the examination of the range of ankle joint dorsiflexion. Elveru et al. [16] and Jonson and Gross [1] report similar ICC values of 0.50 and 0.65. In Part 1 of this study all podiatrists stated that they believed the examination of ankle joint dorsiflexion provided a good indication of dynamic foot function. However, the low reliability and large range of values recorded across assessors questions the clinical value of these examinations. Considering that 10° of dorsiflexion was stated as normal (results from Part 1, based on Root et al. [3], Table 2), clinical measures at either boundary of the 95 % CI (maximum 95 % CI were 5.6° to 15.5°) and the total error of up to 10.7°, could lead to false identification of the actual range of ankle dorsiflexion. This would directly affect the treatment rationale if the outcome suggested limited or adequate range of ankle motion. Moseley and Adams [33] suggest that such variation would make measurement of changes in range of motion due to interventions (e.g. stretching) unreliable. The results from the random effects ANOVA suggest that all three sources of error contribute to variation between assessors. Since random error was quite large (5.2°, left foot, knee flexed), reducing errors from participants and assessors (e.g. through training, use of measurement tools) might still not achieve an acceptable level of reliability.

Classification of first ray mobility demonstrated greater reliability than categorisation of first ray position. The Fleiss Kappa values of less than 0.05 for categorisation of first ray position and range of motion indicate only poor to slight agreement [28,29]. For four of the 12 feet assessed there was greater than 90 % agreement for classification of first ray range of motion as flexible. However, percentage agreement can lack sensitivity as to the true level of agreement between assessors as it can over or under estimate the actual level of agreement and does not account for the possibility that the agreement observed occurred by chance [27]. High levels of agreement for assessment of flexibility might be expected as ‘rigidity’ suggests no motion at all and this is more easily identified than different grades of “some” motion [24]. However, taking into account the Fleiss Kappa and percentage agreement statistical values only poor to moderate reliability was observed. Classification of first ray position demonstrated poor agreement between assessors. There are significant identifiable differences between a plantarflexed and dorsiflexed first ray [24], something that experienced podiatrists would expect themselves to be able to identify. As with measures of rearfoot alignment, first ray position can influence orthotic prescription [24].

Identification of the longer limb provided marginally better agreement than classifying the actual amount of leg length difference, but still only suggests slight agreement [28,29] with Fleiss Kappa values of 0.02 (longer leg) and 0.02 (difference in leg length). This level of reliability is similar to Woerman and Binder-MacLeod [21]. To be able to ascertain that there is a difference in limb length of less than 5 mm requires high precision and it is doubtful that through visual inspection and palpation a clinician could reliably work to such accuracy. If a clinician can identify a discrepancy this small then they will almost always identify a limb length difference because the skeleton is rarely truly symmetrical.

### Clinical implications

One purpose of clinical assessment is to decipher normal from pathological [1,2,23,24] but the results from this investigation suggest that it would not be possible to accurately classify either. The protocol described by Root et al. [3] states precise measurements are required when undertaking a static biomechanical assessment of the foot. Results from this and prior research [11,12,16,32] suggest that such accuracy is not achieved in clinical practice. For example, Root et al. [3,4] states that RCSP and NCSP measurements will precisely dictate the inclination of a rearfoot wedge used in a foot orthoses. However the variability in the assessment of rearfoot position reported here would lead to very different orthotic prescriptions. This directly undermines the biomechanical rationale for intricate adjustments in the design of foot orthoses and the capture of static foot shape as a basis for foot orthosis design. This has profound implications for many areas of clinical practice and suggests a reappraisal of the theoretical and practical basis for orthotic practice is warranted. The low reliability of the assessments evaluated here questions their ability to accurately infer the behaviour of the foot during stance, which is the purpose of the static assessments in the model proposed by Root et al. [4]. Indeed, research investigating the validity of Root et al. [3,4] is currently being undertaken by the authors. The results here also add weight to the case for a move toward objective assessment of dynamic foot behaviour in clinical practice, regardless of the practical challenges this raises.

### Limitations

There are several limitations to the work reported here. Four of the eight examinations used by assessors (from Part 1) were not included in Part 2 of this study. They were excluded because the podiatrists we worked with identified them as ‘optional’. Other clinicians might disagree with the ranking of the eight assessments, especially if their practice is different to that of the podiatrists involved in this current study. Using all eight examinations would have been logistically difficult with the number of assessors and participants in this study and time available for the assessments. The number of assessors used was relatively small and might not represent the true variation across the entire professional communities using the assessments evaluated in this work. All were podiatrists and whilst their professional networks are strongly multi-professional, practices could differ in other disciplines and countries. The literature indicates that the measures used by the assessors and those evaluated in the reliability study, are also used in the physical therapy profession [1,16,20]. The development of the foot assessment protocol occurred through just one iteration of the Delphi method, whereas two or more iterations are often employed [24]. Experience during the exercise suggested that consensus was already in place or very close from the outset. The number of feet assessed was quite small and all participants were free from pathology. The participants were young with an average BMI and may not represent feet that present in many clinical cases. Arguably, assessing these feet is easier than those of people in pain, feet with deformity or in cases of greater BMI, and thus our results might reflect a “best case” scenario in terms of reliability. This study recorded low ICC values, in particular for NCSP and RCSP. The large number of assessors and small number of participants would have increased the variability and therefore could have decreased the inter-assessor reliability. Finally, good reliability does not infer practical usefulness of the assessment. Good reliability may simply reflect low sensitivity and specificity in the measure, or highly repeatable errors by assessors. Thus, good reliability does not infer validity. However, measures cannot be valid unless reliable, and outcomes of this work indicate many of the assessments used in foot health practice are unreliable and thus invalid.

## Conclusions

Static biomechanical assessment of the foot, leg and lower limb is considered important in clinical practice. The key examinations used to make inferences about dynamic foot function, to construct a treatment plan and to determine orthotic prescription are unreliable. Using these examinations to differentiate normal from pathological foot function would not appear to be valid clinical practice.

## Abbreviations

RCSP, Relaxed calcaneal stance position; NCSP, Neutral calcaneal stance position; DF, Dorsiflexed; ASIS, Anterior superior iliac spine; ICC, Values intra-class correlation coefficient values; 95%, CI 95 % confidence interval; SD, Standard deviation; SEM, Standard error of the mean; BMI, Body mass index; MTPJ, Metatarsophalangeal joint; E, Random error of examination variable; E ¹, Assessor error variable; E ² , Subject error variable; ANOVA, Analysis of variance.

## Competing interests

The authors declare that they have no competing interests.

## Authors’ contributions

HJ is the lead researcher and the work forms part of her Doctoral studies. HJ, CN, PB and RJ conceived the research and HJ, CN and PB completed the research design. HJ and PB conducted the data analysis. HJ and CN led on the writing of the paper. All authors read and approved the final manuscript.

## Funding

Funded by University of Salford, School of Health Sciences.

## Supplementary Material

Additional file 1 The questionnaire used for the modified Delphi technique investigation in Phase 1, Part 1. Click here for file
