# Supplementary material for: Inter-assessor reliability of practice based biomechanical assessment of the foot and ankle
Source: J Foot Ankle Res. 2012 Jun 20;5:14. doi: 10.1186/1757-1146-5-14 (PMC3431260; doi:10.1186/1757-1146-5-14)
Supplement: Additional file 1 — The questionnaire used for the modified Delphi technique investigation in Phase 1, Part 1. [file 1757-1146-5-14-S1.docx]

**“An investigation into the biomechanical examination techniques used by Podiatrists in clinical practice.”**

**Please answer the following questions, indicating how you would perform your biomechanical examination of the foot, leg and lower leg during clinical practice.**

**Unless otherwise stated please circle Yes or No or the appropriate answer.**

**Answer all questions anonymously and do no place any form of identification on the questionnaire.**

**No information will be shared with any third parties.**

**This is an investigation into what biomechanical examination techniques Podiatrists use, not an assessment of your skills and practice.**

**Section A**

This section will investigate your use of different examination techniques for assessing the foot and ankle of a patient requiring a biomechanical examination.

**1. a.i. Do you measure Neutral calcaneal stance position (NCSP)? *Yes/No***

a.ii. If you have answered yes to question 1.a.i, how do you measure NCSP?

- Tractograph *Yes/No*

- Goniometer *Yes/No*

- Estimate *Yes/No*

- Other, please state…………………………………………………………………….

a.iii. How often do you use this examination technique on patients requiring a biomechanical

examination?

Please circle the appropriate statement

- Never

- Some of the time

- Most of the time

- All of the time

b.i. Do you measure Relaxed calcaneal stance position (RCSP)? *Yes/No*

b.ii If you have answered Yes, to question 1.b.i, How do you measure RCSP?

- Tractograph *Yes/No*

- Goniometer *Yes/No*

- Estimate *Yes/No*

- Other, please state ……………………………………………………………………

b.iii. How often do you use this examination technique on patients requiring a biomechanical examination?

Please circle the appropriate statement

- Never

- Some of the time

- Most of the time

- All of the time

c. If you have answered Yes to questions 1.a.i and 1.b.i., do you draw a heel bisection line on

the posterior aspect of the calcaneus when performing this assessment? *Yes/No*

d. Do you compare the results of RCSP and NCSP and use this as a measure towards:

d.i. Defining a treatment rationale? *Yes/No*

d.ii. Assessment of foot type? *Yes/No*

**2.a.i. Do you measure the “Forefoot to rearfoot relationship?” *Yes/No***

If you have answered yes to question 2.a.i., please answer the following questions. If you have answered No, please proceed to question 3.

Do you assess the patient:

a.ii. Prone *Yes/No*

a.iii. Supine *Yes/No*

b.i. In what planes do you measure the “Forefoot to rearfoot relationship?”

Please tick in the appropriate boxes.

|  | Yes | No |
| --- | --- | --- |
| Frontal plane |  |  |
| Transverse plane |  |  |
| Sagittal plane |  |  |

b.ii. How do you determine the forefoot relationship in the frontal plane?

- The middle three metatarsals of the forefoot? *Yes/No*

- All five metatarsals of the forefoot? *Yes/No*

c. What classification do you use to measure the “Forefoot to Rearfoot relationship” in

the frontal plane?

c.i. Classify position (e.g Forefoot is parallel to Rearfoot / or Everted /Inverted) *Yes/No*

c.ii. Measure forefoot to rearfoot relationship (e.g 4º Forefoot varus) *Yes/No*

d. How often do you use this examination technique?

Please circle the appropriate statement.

- Never

- Some of the time

- Most of the time

- All of the time

**3.a.i. Do you measure the range of motion at the ankle joint? *Yes/No***

If you have answered yes to question 3.a.i, please answer the following questions. If you have answered No, please proceed to question 4.a.i.

Do you measure the range of motion at the ankle joint:

a.ii. With the knee flexed *Yes/No*

a.iii. With the knee extended *Yes/No*

Do you measure:

a.iv. The total range of motion at the ankle joint? (plantarflexion and dorsiflexion

combined) *Yes/No*

a.v. The range and maximal amount of ankle joint dorsiflexion only? *Yes/No*

Other, please state …………………………………………………………………………

b.i. How do you measure the range of motion at the ankle joint?

- Tractograph *Yes/No*

- Goniometer? *Yes/No*

- Estimate? *Yes/No*

- Other device, please state ………………………………………………………………………

b.ii. How often do you use this examination technique on patients requiring a biomechanical

examination? Please circle the appropriate statement.

- Never

- Some of the time

- Most of the time

- All of the time

**4.a.i. Do you measure the range of motion at the subtalar joint? *Yes/No***

If you have answered yes to question 4.a.i, please answer the following questions, if you have answered No, please proceed to question 5.

Do you assess the patient:

a.ii. Prone *Yes/No*

a.iii. Supine *Yes/No*

a.iv. How often do you use this examination technique?

Please circle the appropriate statement

- Never

- Some of the time

- Most of the time

- All of the time

b.i. Do you establish a subtalar joint neutral position (non-weight bearing) for examining the subtalar joint range of motion? *Yes/No*

b.ii. If you have answered Yes to question 4.b.i, do you palpate the surrounding anatomy of the subtalar joint and move the foot to obtain a neutral position? *Yes/No*

b.iii. Do you assess the direction of motion? (i.e in what plane is motion more evident.) *Yes/No*

c.i. Do you use the Kirby method (“Rotational equilibrium across the subtalar joint axis”) to establish the position of the subtalar joint axis? *Yes/No*

c.ii. Do you establish the “pitch” of the subtalar joint axis in the sagittal plane? *Yes/No*

**5.a.i. Do you measure the range of motion at the midtarsal joint? *Yes/No***

If you have answered yes to questions 5.a.i, please answer the following questions, if you have answered No, please proceed to question 6.

a.ii. How often do you use this examination technique?

Please circle the appropriate statement

- Never

- Some of the time

- Most of the time

- All of the time

b.i. Do you measure the range of motion of the longitudinal axis of the midtarsal joint? *Yes/No*

b.ii. How do you measure the range of motion of the longitudinal axis of the midtarsal joint?

- Tractograph *Yes/No*

- Goniometer? *Yes/No*

- Estimate? *Yes/No*

- Other device, please state ………………………………………………………………………

b.iii. Do you measure the range of motion of the oblique axis of the midtarsal joint? *Yes/No*

b.iv. How do you measure the range of motion of the longitudinal axis of the midtarsal joint?

- Goniometer? *Yes/No*

- Estimate? *Yes/No*

- Tractograph *Yes/No*

- Other device, please state ………………………………………………………………………

c. Do you assess the direction of motion? (i.e in what plane is motion more evident.) *Yes/No*

**6.a.i. Do you measure the range of motion at the 1^st^ Ray? *Yes/No***

If you have answered yes to questions 6.a.i, please answer the following questions, if you have answered No, please proceed to question 7.

a.ii. How often do you use this examination procedure?

Please circle the appropriate statement

- Never

- Some of the time

- Most of the time

- All of the time

b.i. In what planes do you measure the motion of the 1^st^ ray?

Please tick in the appropriate boxes.

|  | Yes | No |
| --- | --- | --- |
| Frontal plane |  |  |
| Transverse plane |  |  |
| Sagittal plane |  |  |

b. How do you quantify this range of motion in the sagittal plane (i.e Dorsiflexion/Plantarflexion)?

b.ii. State in “mm” the range of motion? *Yes/No*

b.iii. Classify it as rigid/flexible/normal? *Yes/No*

Other, please state …………………………………………………………………………

c. Do you assess the position of the 1^st^ Ray? (e.g Determining if it is either plantarflexed/

dorsiflexed/ parallel to the transverse plane of the second metatarsal) *Yes/No*

**7.a.i. Do you measure the range of motion at the 1^st^ metatarsophalangeal joint? *Yes/No***

If you have answered yes to questions 7.a.i, please answer the following questions, if you have answered No, please proceed to question 8.

Do you measure:

b.i. The total range of motion at the 1^st^ metatarsophalangeal joint (Dorsiflexion and Plantarflexion combined)? *Yes/No*

b.ii. The range and maximal amount of dorsiflexion at the 1^st^ metatarsophalangeal joint? *Yes/No*

b.iii. How do you measure the range of motion at the 1st metatarsophalangeal joint?

- Tractograph *Yes/No*

- Goniometer? *Yes/No*

- Estimate *Yes/No*

- Other device, please state …………………………………………………………………….

b.iv. How often do you use this measurements?

Please circle the appropriate statement.

- Never

- Some of the time

- Most of the time

- All of the time

c.i. Do you use “Jack’s test” for assessing the integrity of “The Windlass Mechanism”? *Yes/No*

(Jack’s test: Patient is standing/weight bearing and the hallux is dorsiflexed, the change in the height of the medial longitudinal arch is recorded).

c.ii. How often do you use this measurement?

Please circle the appropriate statement.

- Never

- Some of the time

- Most of the time

- All of the time

**8. a. Do you use the Foot Posture Index? *Yes/No***

If you have answered yes to question 8.a, please answer the following questions. If you have answered no, please proceed to question 9.a.

b.i. Do you use the 8 point Foot Posture Index? *Yes/No*

b.ii. Do you use the 6 point Foot Posture Index? *Yes/No*

c. Why do you use the Foot Posture Index?

c.i. To aid orthotic design and monitor the affect of the orthoses on foot type? *Yes/No*

c.ii. Record a validated measure of foot posture? *Yes/No*

**9.a. Do you use the Foot Health status questionnaire? *Yes/No***

If you have answered yes to question 9.a, please answer the following questions, if you have

answered No, please proceed to **Section B**, question 1.a.

b. Do you use the Foot Health status questionnaire to monitor treatment success? *Yes/No*

c. Do you use it to measure patient perception of their baseline health status? *Yes/No*

d. Other, please state …………………………………………………………………………….

**Section B.**

This section will focus on your assessment of the leg and lower limb.

**1.a. Do you measure and assess the lower limb? *Yes/No***

If you have answered yes to questions 1.a, please answer the following questions, if you have answered No, please proceed to **Section C**.

b. How do you measure for a possible limb length discrepancy?

- Tape measure *Yes/No*

- Palpation of bony landmarks *Yes/No*

- Estimation *Yes/No*

- Other, please state ………………………………………………………………………………..

c.i. Do you measure for an anatomical leg length difference? *Yes/No*

How do you measure for an anatomical leg length discrepancy?

c.ii. Anterior superior iliac spine to medial mallelous? *Yes/No*

c.iii. Posterior superior iliac spine to medial mallelous*? Yes/No*

Other, please state…………………………….………………………………………….

d.i. Do you measure for a functional leg length discrepancy? *Yes/No*

How do you measure for a functional leg length discrepancy?

d.ii. Sternum to medial malleolus? *Yes/No*

d.ii. Umbilicus to medial malleolus? *Yes/No*

Other, please state ……………………………………………………………………….

e. On measuring a limb length discrepancy, how do you record the data?

e.i. To the absolute mm/cm *Yes/No*

e.ii. A range in mm/cm (e.g 1-3cm) *Yes/No*

e.iii. Other, please state………………………………………………………………………….

f. How often do you use this examination technique?

Please circle the appropriate statement.

- Never

- Some of the time

- Most of the time

- All of the time

g. Do you also measure?

g.i. Internal/external Hip rotation? *Yes/ No*

g.ii. The “Q” angle? *Yes/No*

Other, please state……………………………………………………………………………….

**Section C.**

This section will discuss gait analysis.

**1.a. Do you use gait analysis in your biomechanical examination of a patient in clinical practice?**

*Yes/No*

If you have answered yes to question **Section C**.1.a, please answer the following questions, if you have answered No, please proceed to question 2.

b. Do you assess:

- The foot *Yes/No*

- The Ankle *Yes/No*

- The Knee *Yes/No*

- The Hip *Yes/No*

- The Upper body *Yes/No*

**2.**a. Do you have access to and use video/motion capture gait analysis? Yes/No

b. Do you have access to 2D or 3D video analysis equipment? *Yes/No*

c. If you have answered yes to questions 2.a and 2.b, what software do you use?

…...………………………………………………………………………………………………………………………………………………………………………………………………………………

d. How do you interpret/utilise the results from this analysis?

d.i. To aid orthotic design and monitor the affect of orthoses on foot type? *Yes/No*

d.ii. Assess foot and/or lower limb movement? *Yes/No*

Other, please state……………………………………………………………………………………...

e. Do you think having access to this software enhances your examination and treatment plan?

*Yes/No*

**3.**a.i. Do you have access to Pressure plate equipment for gait analysis? *Yes/No*

a.ii. If you have answered yes to question 3.a.i, what equipment/manufacturer do you use?

…………..…………………………………………………………………………………………….

…………………………………………………………………………………………………………

b. How do you interpret/utilise the results from this analysis?

b.i. To aid orthotic design and monitor affect of the orthoses on foot type? *Yes/No*

b.ii. Record a validated measure of foot pressure distribution? *Yes/No*

Other, please state……………………………………………………………………………………...

c. Do you think having access to this software enhances your examination and treatment plan?

*Yes/No*

**4**.a.i. Do you have access to Force plate equipment for gait analysis? *Yes/No*

a.ii. If you have answered yes to question 4.e.i, what equipment do you use?

…………...…………………………………………………………………………………………….

…………………………………………………………………………………………………………

b. How do you interpret/utilise the results from this analysis?

b.i. To aid orthotic design and monitor affect of orthoses on foot type? *Yes/No*

b.ii. Record a validated measure of force and calculate moments at joints? *Yes/No*

Other, please state……………………………………………………………………………………...

c. Do you think having access to this software enhances your examination and treatment plan?

*Yes/No*

**5.** Please identify 5 key events (e.g Position of heel at initial contact) of the gait cycle that you would analyse when conducting a clinical gait analysis assessment.

1.

2.

3.

4.

5.

**6.** Please state any other biomechanical assessment you would use?

………………………………………………………………………………………………………………………………………………………………………………………………………………………………………………………………………………………………………………………………………………………………………………………………………………………………………………………………………………….………………………………………………………………...

**7.** Additional comments:

…………………………………………………………………………………………………………………………………………………………………………………………………………………………………………………………………………………………………………………………………………………………………………………………………………………………………………………………………………………………….……………………………………………………...
